# ImmuCellDB: An Indicative Database of Immune Cell Composition From Different Tissues and Disease Conditions in Mouse and Human

**DOI:** 10.3389/fimmu.2021.670070

**Published:** 2021-08-12

**Authors:** Ziyi Chen, Han Na, Aiping Wu

**Affiliations:** ^1^Institute of Systems Medicine, Chinese Academy of Medical Sciences & Peking Union Medical College, Beijing, China; ^2^Suzhou Institute of Systems Medicine, Suzhou, Suzhou, China; ^3^Department of Infectious Diseases, The Second Hospital of Nanjing, The Affiliated Hospital of Nanjing University of Chinese Medicine, Nanjing, China

**Keywords:** immune cell, deconvolution, human, mouse, transcriptome, database

## Abstract

Immune cell composition is highly divergent across different tissues and diseases. A comprehensive resource of tissue immune cells across different conditions in mouse and human will thus provide great understanding of the immune microenvironment of many diseases. Recently, computational methods for estimating immune cell abundance from tissue transcriptome data have been developed and are now widely used. Using these computational tools, large-scale estimation of immune cell composition across tissues and conditions should be possible using gene expression data collected from public databases. In total, 266 tissue types and 706 disease types in humans, as well as 143 tissue types and 61 disease types, and 206 genotypes in mouse had been included in a database we have named ImmuCellDB (http://wap-lab.org:3200/ImmuCellDB/). In ImmuCellDB, users can search and browse immune cell proportions based on tissues, disease or genotype in mouse or humans. Additionally, the variation and correlation of immune cell abundance and gene expression level between different conditions can be compared and viewed in this database. We believe that ImmuCellDB provides not only an indicative view of tissue-dependent or disease-dependent immune cell profiles, but also represents an easy way to pre-determine immune cell abundance and gene expression profiles for specific situations.

## Introduction

Tissues infiltrating immune cells have long been recognized as important regulators in both healthy and disease conditions. In response to different stimuli, normal and abnormal immune reactions may be produced by the immune system. For instance, autoimmune diseases can occur when the immune reactions targeting our body are too strong, whereas tumors can be established when immune responses to malignant cells are too weak. When fighting external pathogens, inflammation or infection can occur depending on the magnitude and duration of immune responses (https://www.budandtender.com/blogs/bud-tender-blog/your-endocannabinoid-and-immune-system). In addition to local immune responses, systematic multi-organ immune responses frequently happen in many diseases. Immune states in multiple irrespective areas can also be reshaped by some cytokines, metabolites, etc., that are transported by the circulatory system (1). Therefore, knowledge of the constitution of tissue immune cells under different conditions should greatly enhance our understanding of their roles.

Usually, tissue immune cell abundance is measured using well-known methods including flow cytometry (2), immunochemistry (3), etc. However, these experimental-based procedures are usually conducted in a laboratory and are time-consuming when batch processing many biological samples. Additionally, cell type–specific markers and corresponding antibodies are not readily available in many circumstances. Although some public databases of flow cytometry data like Immport (4) or FlowRepository (5) offer users access to download experimental data corresponding to a specific study, the number of tissue and disease categories is still small and may restrict researchers from querying tissue immune cell abundances they are interested in. Recently, with the advancement of high-throughput transcriptome measuring technologies, multiple computational tools have already been designed and used to study the abundance of tissue immune cells in terms of omics data, including DNA microarrays, RNA-seq, and DNA methylation, etc. (6, 7) The suitable performance of these computational-based methods has been validated in multiple studies. Compared to an experimental based strategy, tissue immune cell composition can be rapidly estimated from genomics data. Additionally, tissue transcriptome data from most tissue and disease types has already been deposited into some public database like Gene Expression Omnibus (GEO) (8). These represent a great resource for researchers for transcriptome data under different conditions (9, 10).

However, there are still no available web database search engine for users to query the differences in abundance of tissue immune cells between different tissue and disease types. With tissue expression data accumulated in GEO, an in-depth knowledge of the inner immune cell constitution allows easy prediction from tissue expression data. Therefore, predicting the composition of tissue immune cells from tissue transcriptome data should greatly accelerate our understanding of the dynamics of immune cells in different situations. Furthermore, a user-friendly database interface covering all possible tissue and disease combinations will be convenient for those seeking to view the landscape of tissue immune cell abundance under different situations.

Thus, by mining the transcriptome data profiles from microarray data from both humans and mice in GEO, transcriptome data including nearly 266 tissue types and 706 disease types in humans and 143 tissue types, 61 disease types, and 206 genotypes from mouse were collected. Next, using immune cell estimation tools developed for human and mouse, the composition of 21 immune cells was calculated. By integrating resources for immune cell composition and gene expression across different tissue and disease conditions into this database, users can browse, search, and view data in accordance with the selection items provided. This database represents an indicative resource for tissue immune cell compositions over tissue types, diseases, and genotypes, and can provide a convenient querying tool for other researchers.

## Materials and Methods

### Data Collection and Preprocessing

**Figure 1:**
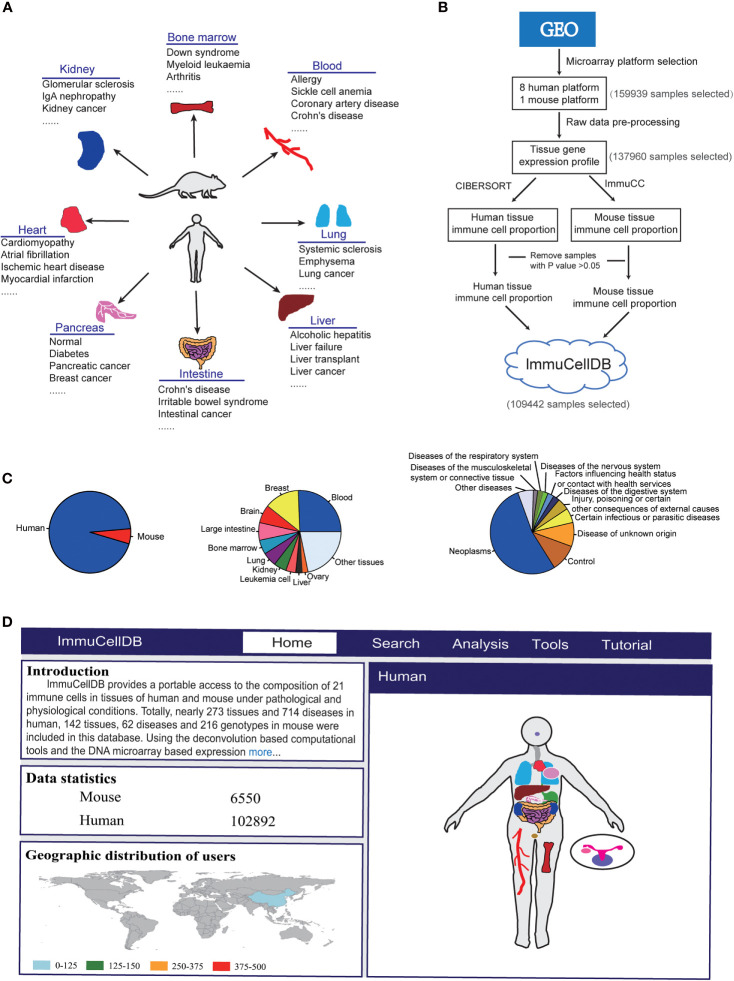
Schematics for ImmuCellDB. **(A)** A brief introduction for the relationship of tissues and diseases in the database. **(B)** The workflow for the construction of database. Modules for data collection, data preprocessing, immune cell estimation and database construction are shown. **(C)** A pie plot for the relative proportion of species and top 10 most popular tissues and diseases. **(D)** A screenshot for the homepage of ImmuCellDB. The home page was consisted of four parts that is the introduction for the database, statistics of sample data, the worldwide distribution for all users to this database and a body map of human and mouse organs.

ImmuCellDB aims to provide an indicative landscape of tissue immune cell composition across different tissue and disease conditions (**Figure 1A**). All human and mouse transcriptome data were mined from GEO with the R package “GEOmetadb” (11). The cell lines, Image result for flow cytometry sorted or magnetic bead enriched cell types, and some synthetic mixtures were filtered out based on their characteristics, title, and source name for each Sample accessions numbers (GSM). Finally, a summary table including GSM IDs, GSE IDs, Platform name, and sample description content (the title, source name and characteristics of GSM and the summary and the overall design part of GSE for each sample) were tabulated (**Supplementary Figure 1**).

Then, the characteristics for each sample including tissue type, disease type, and genotype were manually extracted from the sample description contents. As there were many different forms of names for some tissues, diseases, and genotypes, samples under the same conditions may have been misclassified into different groups. Therefore, a standardized disease name was helpful for avoiding confusions made by different disease nomenclature. Here, all disease types collected from GEO were manually retrieved from International Classification of Diseases version 11 (ICD-11) and unified according to the disease classification rules in this database.

With respect to all these selected samples, the raw CEL files were downloaded and samples profiled on the sample microarray platforms were processed with the same pipeline (**Figure 1B**). The probsets for each microarray platform were annotated with the help of some custom CDF packages (http://brainarray.mbni.med.umich.edu/Brainarray/Database/CustomCDF/). All scripts used to process raw data can be found on Github (https://github.com/wuaipinglab/ImmuCC/tree/master/ImmuCellDB/). Then, expression matrices for different microarray platforms were merged into one large matrix according to the common genes shared by them. To ensure that the expression levels were distributed in a similar range, a quantile normalization method was used for each dataset. In addition, gene IDs from mouse expression data were converted into the orthologous human gene IDs.

### Immune Cell Proportion Estimation

Using processed gene expression data, tissue immune cell compositions across all different conditions could be calculated with some previously reported deconvolution tools designed specifically for microarray data (**Figure 1B**) (6). For human samples, the relative proportion of 22 immune cells was estimated with CIBERSORT (9). With respect to the mouse transcriptome data, the composition of 25 immune cells was predicted with our own model array_ImmuCC (10). To help the users view the marker genes selected for each immune cell, a matrix for the relationship between each immune cell and all signature genes was available at the **Supplementary Table 1**. The signature genes for human immune cells were directly downloaded from the **Supplementary Table 1** of CIBERSORT(https://static-content.springer.com/esm/art%3A10.1038%2Fnmeth.3337/MediaObjects/41592_2015_BFnmeth3337_MOESM207_ESM.xls) (9). The signature genes for mouse immune cells were derived from our previous research (10). Samples with P values > 0.05 were discarded as this may reflect a lower immune cell infiltration (12).

### Categorization of Different Tissue Diseases and Immune Cells

To facilitate viewing of results, a tree browser for different tissue and disease types was constructed. First, all collected tissue names were unified according to their anatomical location. The tissues were then hierarchically organized into three levels. Next, to keep the consistency across all disease types, name unification was also manually performed for all disease names. The corrected disease names were then manually searched from ICD-11 (https://icd.who.int/browse11/l-m/en). Both the disease classification in ICD-11 and the GEO characteristics of data were taken into consideration, and all samples were then grouped into a corresponding disease tree. Firstly, since the disease names of samples provided in GEO were not the standard names, it is hard for us to directly put them into the correct positions in the ICD disease classification tree. At first, we had unified the disease names with reference to ICD-11 and prior knowledge. For a disease with multiple disease names, these disease names were replaced with the correspondent disease name listed in ICD-11. For instance, both Breast carcinoma, Mammary gland tumor and Breast tumor, Breast cancer were grouped into Breast cancer. For some disease names used in GEO while not existed in ICD-11, the original disease names from GEO were kept. For instance, the prostate cancer was classified as Adenocarcinoma of prostate (2C82.0), Other specified malignant neoplasms of prostate (2C82.Y) and Malignant neoplasms of prostate, unspecified (2C82.z) in ICD-11. In ImmuCellDB, the prostate cancer was grouped as Adenocarcinoma of prostate, prostate cancer and non-metastatistic prostate cancer. Except for Adenocarcinoma of prostate, the other two diseases were not described in ICD-11 which were kept in ImmuCellDB. Another question is that too many hierarchical branches of diseases are not convenient for searching. To address this issue, the disease classification tree provided in our database was only consisted of three levels. We classified all diseases into groups according to the top-level of the disease classification tree in ICD-11. For instance, for certain infectious or parasitic diseases, we put all diseases including HIV infection, Leprosy, Influenza infection into this group. Then, diseases were merged into the second level according to prior knowledge. With respect to HIV infection, the group was finally consisted of multiple disease names including HIV infection, HIV-1 infection, HIV-1 subtype C infection, etc. Finally, to ensure the comparability of the immune cell types predicted between these two species, immune cell types calculated with these two computational models were unified according to their location in our immune cell differentiation tree. Specifically, they were aggregated in the following ways. For human immune proportion data, Mast cells = Mast cells resting + Mast cells activated, and T cells CD4 activated = T cells CD4 memory activated. For mouse immune proportion data, T cells CD8 = T cells CD8+ T cells CD8 + T cells CD8, T cells CD4 activated = Th1 Cells + Th2 Cells + Th17 Cells, and Dendritic cells resting = Dendritic cells immature. Finally, immune cells predicted by these two models were merged into 21 types including: Mast cells, Neutrophils, Eosinophils, Monocytes, Macrophages M0, Macrophages M1, Macrophages M2, Dendritic cells activated, Dendritic cells resting, B cells memory, B cells naive, Plasma cells, T cells CD4 naive, T cells CD4 memory, T cells follicular helper, Tregs, T cells CD4 activated, T cells CD8, Gamma delta T cells, NK cells resting, and NK cells activated.

### Web Server Construction

ImmuCellDB was implemented using the MongoDB system. The metadata for species, tissues, diseases or genotypes from each sample were stored in the MongoDB database with a unique key ID. The web interface of this database was constructed based on the Node.js framework, which comes with an Application Programming Interface of the MongoDB built on top of JavaScript libraries including jQuery, Bootstrap, ECharts, Highcharts and additional plugins. All figures and tables were generated with R scripts.

### Data Access

To enable quick access of the data deposited in this database, all transcriptome and immune cell composition data are also available at Figshare (https://figshare.com/articles/dataset/ImmuCellDB/13546529). All transcriptome data used in ImmuCellDB were collected from public database and will not be used for other commercial purpose.

## Results

### Database Summary

ImmuCellDB is a database for tissue immune cell composition and gene expression in nearly 266 tissue types and 706 disease types in humans and 143 tissue types, 61 disease types, and 206 genotypes in mouse (**Figure 1C**). According to the expression data collected from GEO, the abundance of 21 different types of immune cells was inferred from the transcriptome data of tissues using deconvolution tools. To reduce the display space, tissue types and disease types were categorized into some major groups. ImmuCellDB contains five major modules: home, search, analysis, tools and tutorial (**Figure 1D**). From the home module, users can view the introduction for this database, the worldwide distribution of users and the sample number for each tissue. By clicking each tissue in the body of human and mouse, all samples belong to this tissue will be selected. The search module gives an access to tissue immune cell compositions over certain tissue types, disease types or genotypes. The analysis module is consisted of four tools: ImmDiff, ExpresDiff, Correlation and NetCor. The variation and correlation of immune cell abundance and gene expression level between different conditions can be compared and viewed with these tools. An overview for the functions and results of the search module and analysis module were indicated at **Supplementary Figure 2**. The tools module allows users to estimate the composition of tissue immune cell with three our previously reported tools: Array_ImmuCC (10), Seq_ImmuCC (13) and Tissue_ImmuCC (14). The tutorial module provides an introduction for search, analysis and tools. Also, a worked example of how to find a valuable information from this database was also available at the **Supplementary Materials**.

### Search Module

**Figure 2:**
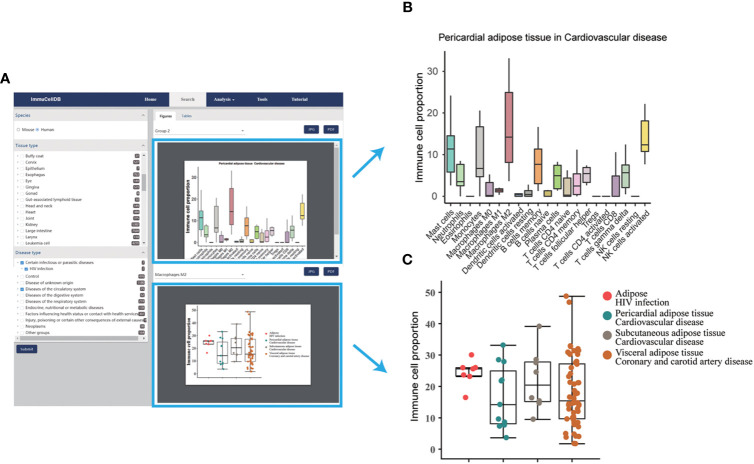
A snapshot for search module. **(A)** Result page for the search module. The selection box for species, tissues and diseases was located on the left side of this page and all figures and tables generated were listed in the right side of this page. **(B)** Boxplot for the proportions of 21 immune cells under the selected tissue and disease conditions. Notably, only the first 10 tissue and disease condition groups was displayed. **(C)** Boxplot and dotplot for the proportion of each immune cell type across different conditions. One immune cell type correspondent to one figure.

ImmuCellDB provides a tree browser for users to query for tissue immune cell compositions over certain tissue types, disease types or genotypes using the search module. As indicated in **Figure 2A**, by sequentially selecting the species, tissue type, disease type or genotype from the drop-down box, two figure types plus a table will be returned to the result page. From the image figure box, a box plot for the relative proportion of all 21 immune cells in each condition was generated (**Figure 2B**). Next, to illustrate the variation for each immune cell over different conditions, the proportional distribution of each immune cell across all selected conditions was displayed with a boxplot (**Figure 2C**). In some situations, users may want to integrate this retrieved information with other data. To meet these special requirements, the table consisted of the sample information including GSM IDs, tissue types, disease types or genotypes, and the abundance of 21 immune cells. Users can download all these figures and tables directly from this result page.

### Analysis Module

#### ImmDiff Tool

**Figure 3:**
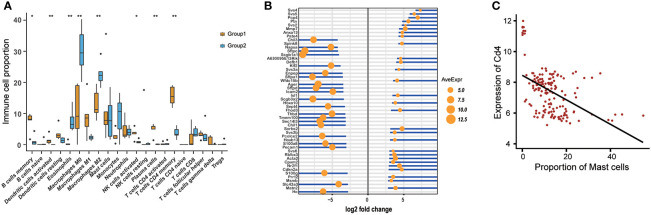
Results from the analysis modules in ImmuCellDB. **(A)** The variation of immune cell abundances between two groups returned by ImmDiff tool. A comparison for the proportional difference of each immune cell between the two groups was conducted with a t-test and the P-value was used to interpret the difference (**indicates the P-value < 0.01, * means 0.01 < P-value < 0.05). **(B)** Confects plot for the differentially expressed genes between two conditions by ExpresDiff tool. The area of the dot was proportional to the averaged gene expression value and the length of the line was negative correlated with the log2 fold change. **(C)** Dot plot for the correlation between the immune cell abundance and gene expression by Correlation tool. The glm method in geom_smooth function of “ggplot2” package was used to fitting for data points.

Many factors including species, tissue types, disease types, or genotypes may have an impact on the context of tissue immune cells. To evaluate the differences in tissue immune cell levels, the ImmDiff tool in the Analysis function was designed to compare the immune cell proportion between two specified condition groups. Based on the two groups selected from the selection box covering species, tissue type, disease type, or genotype, a box plot was used to show the distribution of all immune cells between these two groups (**Figure 3A**). Moreover, a comparison between these two groups was carried out with a t-test, and the immune cells with significant differences in proportion were marked with asterisks. Furthermore, users can compare their own data with the samples collected in this database using the “Comparing with uploaded data” module. The same result format will be generated under this selection. Based on this analysis, immune cells with different abundance can be successfully identified.

#### ExpresDiff Tool

Gene expression levels also vary across different species, tissue types, disease types, and genotypes. Knowledge of the differentially expressed genes under different conditions is useful for understanding the divergence of transcriptome levels. The ExpresDiff tool developed here allows users to identify the gene expression changes between different condition groups. After setting two biological conditions in the selection box, the gene expression difference between these two groups could be obtained. The differential expression analysis was conducted using a confect ranking method with the “topconfects” package and ranked according to the confident effect size (15). Users can download the whole table directly from the result page. Then, a plot representing the fold change and averaged expression level of genes with the highest effect size was generated (**Figure 3B**). Additionally, to investigate the expression pattern of the most differentially expressed genes, the expression of the top 50 genes over all samples belonging to these two groups was exhibited using a heatmap. Similar to the ImmDiff tool, users can also compare their own data with the samples deposited in this database using the “Comparing with uploaded data” module. It should be noted that comparison of the gene expression differences between human and mouse is not suggested here.

#### Correlation Tool

The correlation between immune cell abundance and gene expression values may reflect a possible direct or indirect relationship between genes and immune cells. For each cell type, the significantly correlated genes may relate to their character, such as differentiation stage and functional roles. For instance, a positive correlation between T cells CD8 and PD1 was associated with the dysfunction of CD8 T cells (16). The Correlation tool designed here offers access for users to easily view the correlation between genes and immune cells. With samples in the selected species, tissue types, disease types, cell types, and gene symbols, the Spearman correlation between gene expression level and immune cell abundance was calculated. A dot plot was used to illustrate the correlation between each immune cell and gene pairs across all selected samples (**Figure 3C**). A linear fitting was also added to the dot plot to help interpret the tendency across all data sets.

#### NetCor Tool

With the Correlation tool described above, analysis of the correlation between a small amount of genes and immune cell pairs is also possible. However, a comprehensive picture of the correlation between immune cell abundance and gene expression values can provide an important resource for their inner relationship in distinct tissue microenvironments. The NetCor tool makes this analysis possible by integrating all correlated immune cell and gene pairs into a table. For all selected data sets, the Spearman correlation between the expression level of each gene and the abundance of each immune cell was calculated. Finally, all immune cell types and gene pairs whose correlation efficiency exceeds 0.7 were returned to the result page. The results are displayed in a tabular format, with each row representing a correlated immune cell type and gene pairs, as well as a correlation coefficient.

## Discussion

We report here the ImmuCellDB database, which provides access to the immune cell proportion and gene expression data from over 266 tissue types and 706 disease types in human and 143 tissue types, 61 disease types, and 206 genotypes in mouse. Utilizing the results estimated from tissue transcriptome data, ImmuCellDB provides a collective resource for tissue immune cell abundances and gene expression profiles from different conditions.

Using ImmuCellDB, the distribution of the relative proportion of 21 immune cells in each tissue and disease combinations, and the variation in the distribution of each immune cell over different tissue and disease groups can be viewed with the Search module. Limited by the size of the web page, only the first 10 selected tissue and disease groups are used to generate a plot when the number of tissue and disease combinations is larger than 10. In addition to searching from the database, tissue immune cell abundance and gene expression profile differences between different tissues and disease conditions can be calculated. The immune cells with different abundance and the genes with significantly different expression levels between these two groups can be calculated with ImmDiff and ExpresDiff. This information will support an understanding of the different immune reaction pathways and gene transcription profiles between any two selected conditions. Besides an analysis of immune cells and genes separately, the correlation between any immune cell and gene pairs can also be defined according to the Spearman correlation between cell abundance and gene expression level. With the Correlation and NetCor tools, users can obtain the correlation profile of immune cells and genes under any specified conditions. It should be noted that a high correlation coefficient was not equal to a participation of a gene in a cell’s function.

However, all datasets used in this database were derived from DNA microarray-based transcriptome platforms because more immune cell types were included in computational tools designed specifically for microarray data. Additionally, multiple methods have been developed to get the abundance of tissue immune cells from other omics data, including bulk RNA-seq (13), bisulfite sequencing (17), and ATAC-seq (18). Leveraging these sequencing-based data resources, we can estimate the immune cell counts from other bulk tissue omics data. In addition to bulk studies, some single cell detecting methods such as single cell RNA-seq (19) and mass cytometry (20) can also be used to determine cell amounts. By assigning each cell to the correspondent cell type, the proportion of each cell population can be successfully enumerated (21). Therefore, further integrating the immune cell abundance estimated from multi-level omics data into this database should expand the application of ImmuCellDB to additional tissues and disease categories.

Altogether, ImmuCellDB is a valuable resource for researchers to study the immune cell abundance and gene expression levels of tissues in different conditions.

## Data Availability Statement

The original contributions presented in the study are included in the article/**Supplementary Material**. Further inquiries can be directed to the corresponding author.

## Author Contributions

ZC and AW conceived and designed the study. ZC and AW analyzed the data and results. HN contributed to the discussion and analysis of the studies. ZC and AW wrote the paper. All authors contributed to the article and approved the submitted version.

## Funding

This work was supported by: 1 The National Key Plan for Scientific Research and Development of China (2016YFD0500301), 2 The CAMS Initiative for Innovative Medicine (2016-I2M-1-005), 3 The Six-talent Peaks Project in the Jiangsu Province (SWYY-169), 4 The Open Project Program of the National Laboratory of Pattern Recognition (NLPR) (201900004), 5 The Non-profit Central Research Institute Fund of Chinese Academy of Medical Sciences (2018RC310022), 6 Central Public-Interest Scientific Institution Basal Research Fund (2016ZX310195, 2017PT31026 and 2018PT31016).

## Conflict of Interest

The authors declare that the research was conducted in the absence of any commercial or financial relationships that could be construed as a potential conflict of interest.

## Publisher’s Note

All claims expressed in this article are solely those of the authors and do not necessarily represent those of their affiliated organizations, or those of the publisher, the editors and the reviewers. Any product that may be evaluated in this article, or claim that may be made by its manufacturer, is not guaranteed or endorsed by the publisher.

## Supplementary Material

Supplementary Figure 1 An overview for transcriptome datasets selection and filtering process.

Supplementary Figure 2 An introduction for the functions and results of Search module and the Analysis module.

Supplementary Table 1 Matrix for the relationship between each immune cell and all signature genes. In this table, the marker genes were indicated as ‘1’ and the remaining genes were indicated as ‘0’.

Supplementary Materials Three simple work example on how to find a valuable information from this database.
