# Supplementary material for: ImmuCellDB: An Indicative Database of Immune Cell Composition From Different Tissues and Disease Conditions in Mouse and Human
Source: Front Immunol. 2021 Aug 12;12:670070. doi: 10.3389/fimmu.2021.670070 (PMC8387820; doi:10.3389/fimmu.2021.670070)
Supplement: Supplementary Figure 1 — An overview for transcriptome datasets selection and filtering process. [file DataSheet_1.docx]

**Supplementary Figures**

**Supplementary Figure 1:** An overview for transcriptome datasets selection and filtering process.

**
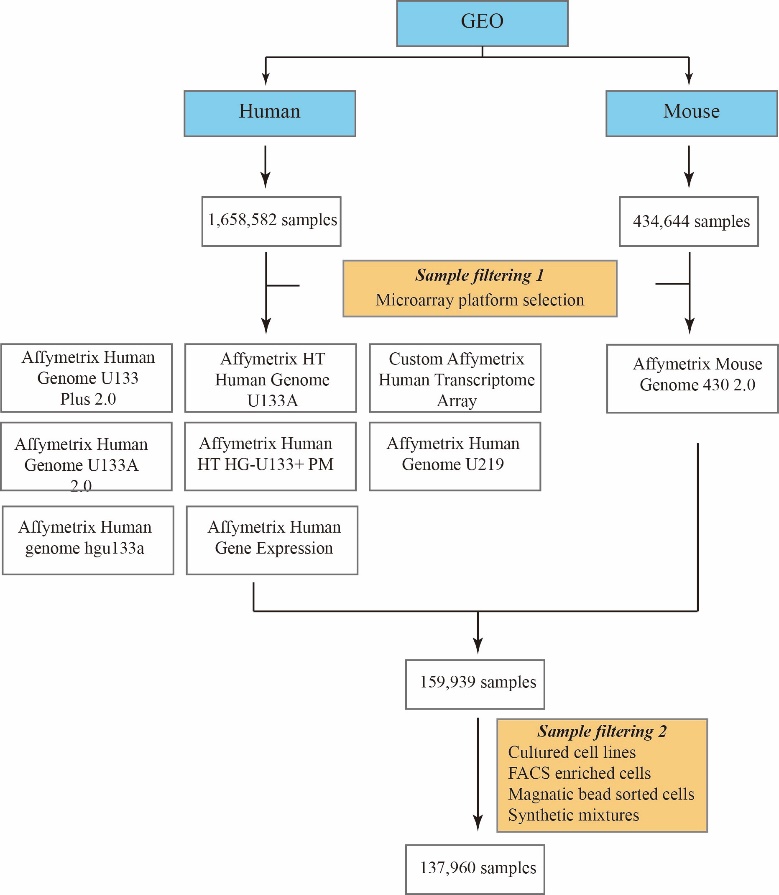
**

**Supplementary Figure 2:** A brief introduction for the functions and results of Search module and the Analysis module.

**
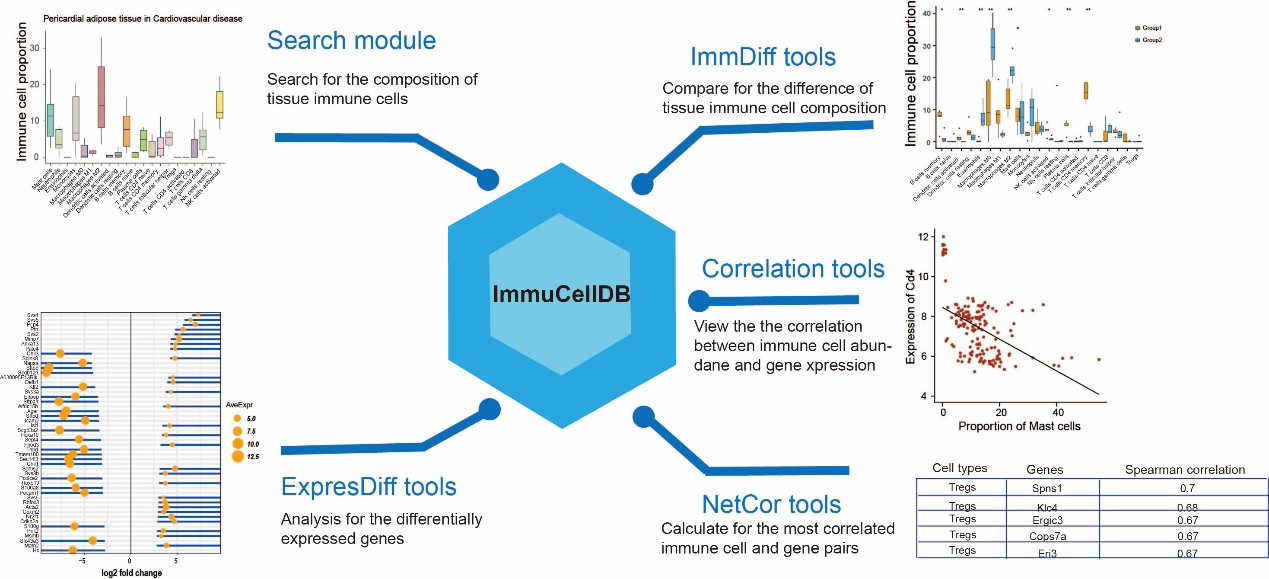
**

**Application cases with ImmuCellDB**

**1, Evaluate for the composition of blood immune cells in HIV infected and non infected controls with “*Search*” module.**

Tissue immune cell composition can vary across different tissues and diseases. With the “*Search*” module, we can view the composition of blood immune cells before and after infected with HIV. By selecting for the species, tissues and diseases from the selection box, the relative proportion of different immune cells across different tissues and disease conditions will be displayed in the result page. Here, the species to “Human”, the tissues types to “Blood_cell_Leukocyte_Lymphocyte_PBMC” and the diseases correspondent to HIV including “Chronic HIV infection_HIV infection_HIV positive_HIV positive with invasive pneumococcal disease_HIV resistant_HIV-1 infection_HIV-1 subtype C infected_HIV/HCV coinfected_Putative progressor HIV_Normal” were selected. By clicking the “submitting” button, the picture for the composition of immune cells in blood, leukocyte, lymphocyte and PBMC under different HIV status was returned. As indicated in **Figure 1** and **Figure 2**, a relative higher proportion of CD8 T cells and lower proportion of CD4 T cells was observed in HIV-1 infected samples when compared with the healthy individuals.


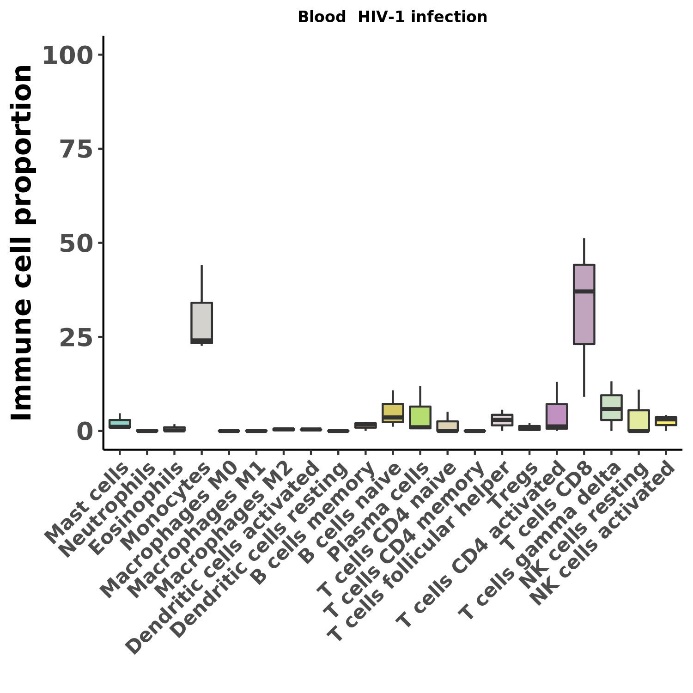


**Figure1.** The relative proportion for 21 immune cells in HIV-1 infected blood.


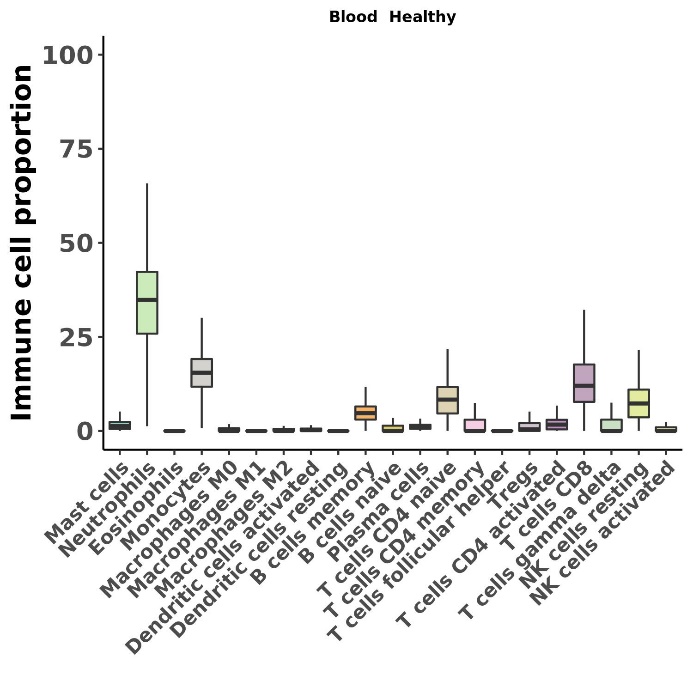


**Figure2.** The relative proportion for 21 immune cells in healthy blood samples.

**2. Assess for the immune cell difference between Chronic HIV infection and normal individuals with “*ImmDiff*” tool.**

Except view the difference of immune cell constitution from the separated figures returned by the Search module, the difference for tissue immune cell abundance can also be directly evaluated with the **“*ImmDiff*”** tool. Here, in ***ImmDiff***, by selecting for the species, tissues and disease in Group 1 as “Human”, “Blood”, “Chronic HIV infection” and the correspondent items for the Group 2 was selected as “Human”, “Blood”, “Normal”. As shown in **Figure 3** below, a boxplot for the difference of immune cell proportion between these two groups was obtained. Consistent to our prior knowledge, a significant higher proportion of CD8T cells and relative lower proportion of CD4 T cells was observed. Besides, neutrophils are the most abundant leukocytes in all selected blood samples.


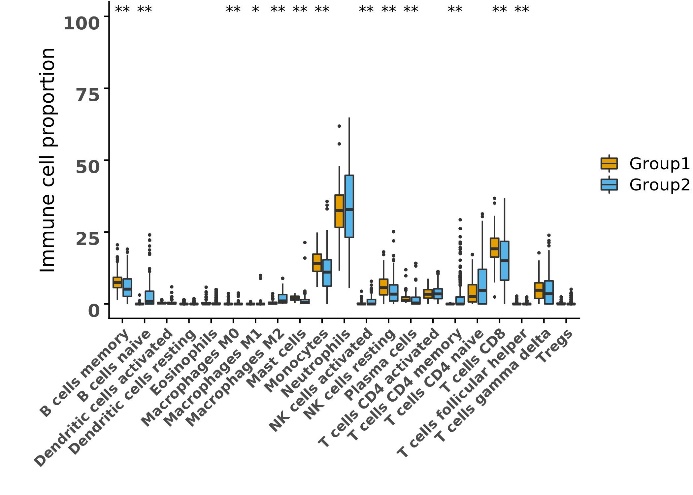


**Figure 3.** Boxplot for the immune cell proportion difference between the HIV infected individuals(Group1) and normal individuals(Group2) calculated by “***ImmDiff***”.

**3. Analyzing for gene expression difference between different conditions with** **“*ExpresDiff*” tool.**

In addition to assess for the composition of tissue immune cells, the differentially expressed genes(DEGs) between different tissue and disease conditions can also be obtained with our database. Here, in **“*ExpresDiff*”** tool, by selecting for the species, tissues and disease in Group 1 as “Human”, “Blood”, “Control” and the items for the second group was selected as “Human”, “Breast”, “Control”. Here, 760 sample belong to Group1 and 27 sample in Group2 was extracted from the database and the DEGs were calculated with a confect ranking method in “topconfects” package. As indicated in **Figure 4**, the figure to the fold change and the averaged expression level for the top 50 DEGs was generated. Besides, the expression for this top 50 DEGs in all selected samples(760 sample belong to Group1 and 27 sample in Group2) were illustrated with a heatmap(**Figure 5**).


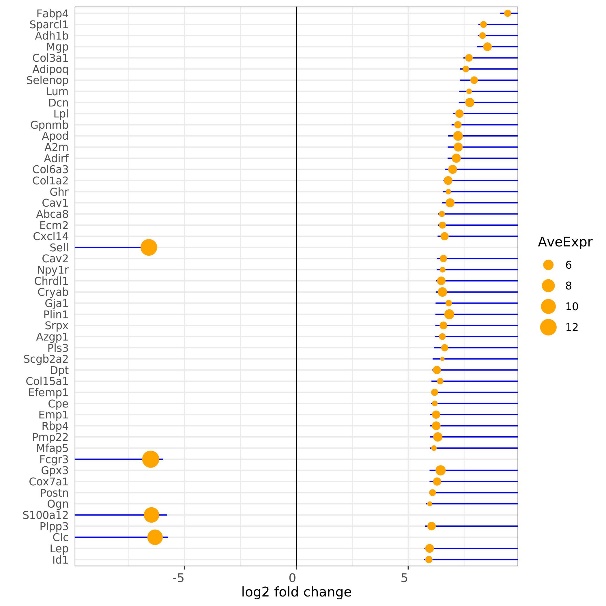


**Figure4.** Confects plot for the fold change and averaged expression value of the top 50 DEGs calculated between human blood and breast tissues.


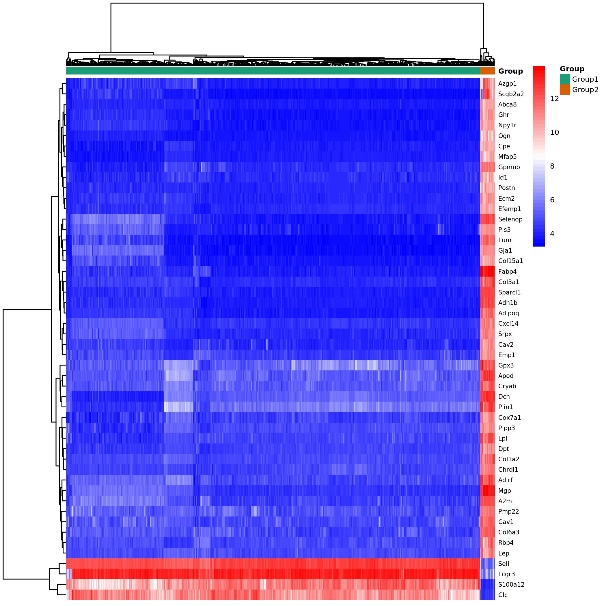


**Figure 5.** Heatmap plot for the expression of top 50 DEGs in all selected human blood and breast tissue samples.
